# Facial morphology predicts male fitness and rank but not survival in Second World War Finnish soldiers

**DOI:** 10.1098/rsbl.2013.0049

**Published:** 2013-08-23

**Authors:** John Loehr, Robert B. O'Hara

**Affiliations:** 1 Lammi Biological Station, University of Helsinki, Pääjärventie 320, Lammi 16900, Finland; 2 Department of Biosciences, Ecological Genetic Research Unit, University of Helsinki, PO Box 65, Helsinki 00014, Finland; 3 Biodiversity and Climate Research Centre, Senckenberganlage 25, 60325 Frankfurt am Main, Germany

**Keywords:** lifetime reproductive success, social dominance, survival, war, facial morphology

## Abstract

We investigated fitness, military rank and survival of facial phenotypes in large-scale warfare using 795 Finnish soldiers who fought in the Winter War (1939–1940). We measured facial width-to-height ratio—a trait known to predict aggressive behaviour in males—and assessed whether facial morphology could predict survival, lifetime reproductive success (LRS) and social status. We found no difference in survival along the phenotypic gradient, however, wider-faced individuals had greater LRS, but achieved a lower military rank.

## 1. Introduction

A constant feature throughout human evolution has been intraspecific conflict [1–3], and simulations have identified warfare as a possible major component of human social evolution [2]. Aggressive individuals who have participated in small-scale warfare and revenge killings may receive fitness costs [4] or benefits [5]. While these two previous studies allow some inferences to be made about small-scale tribal warfare, little is known of the survival and fitness benefits for individuals in large-scale conflicts.

**Figure 1.**
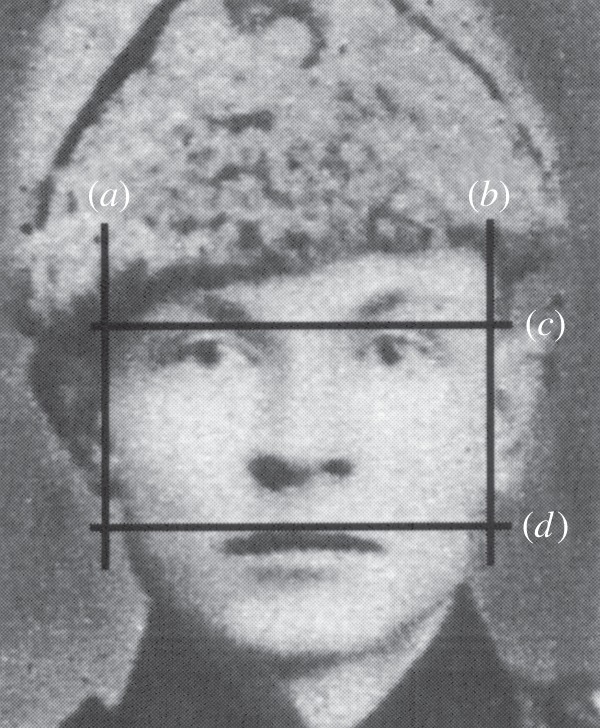
Example of measurements of fWHR as upper bizygomatic width (line *a*–*b*) versus facial height (*c*–*d*) in a fallen Finnish soldier. (available at source)

Facial morphology provides a particularly useful proxy measure for male behaviour, and the wealth of historical photographs and data available allow hypotheses essential to the study of human evolution to be tested. Research has demonstrated that male facial morphology can predict social dominance [6], sexual attractiveness [7,8], reproductive success [9,10], testosterone levels [11] as well as strength and fighting ability [12]. In particular, the facial width-to-height ratio (fWHR, [13]; figure 1) predicts a suite of characters in males: aggressiveness [14,15], mortality in violent conflicts [16], cooperative ability [17] and trustworthiness [18,19]. Wider-faced men have higher testosterone levels [20], are more aggressive ([14,15]; but see [21,22]), and are perceived by others to be more aggressive [23]. Wider-faced males exploit trust more often, and others tend to trust thinner-faced males more readily [18,19], however, in the presence of competition, wider-faced males have also been shown to demonstrate greater cooperation with peers [17].

On 30 November 1939, the Soviet Red Army invaded Finland, starting the 3.5 month long Winter War. We used Finnish archives to collate data on survival and number of offspring for soldiers who fought in this war to explore the correlation of fWHR with rank, fitness and survival. Mueller & Mazur [6] found that dominance scores given to faces of recruits in an elite US military academy predicted future rank and lifetime reproductive success (LRS), but not survival during conflicts. Previous research has shown that fWHR predicts mortality with wider-faced males less likely to die violent deaths, but only when close physical contact is involved, and not when technology is used [16], and a positive relationship between fWHR and LRS has been found in a historic Austrian population [24].

## 2. Material and methods

To enable comparison of soldiers with a similar probability of survival, data (available via doi:10.5061/dryad.d5vh1) were primarily gathered from two infantry regiments (JR 16, *n =* 333 and JR 21, *n =* 312), and one artillery regiment (KTR 3, *n* = 60). These regiments were selected based on the availability of photographs from the sources listed below. Photographs of soldiers who died during the war (*n* = 510) were scanned from their source [25]. Birth and death dates, birth place, rank, regiment, marital status and number of children were found for these soldiers from the online database of the National Archives of Finland (http://kronos.narc.fi/menehtyneet/). Data for surviving soldiers (*n =* 285) were scanned from their source [26]. Scanning focused on the three regiments, however, scanned pages that contained soldiers from other regiments (*n* = 90) were included in analysis. Records include photographs, military rank, birth date and place, regiment(s) served with and birth date of children. For analysis, rank at the start of the Winter War was divided into three categories: enlisted ranks (ranks below officers), junior officers and senior officers. See the electronic supplementary material, S1 for more information on these data.

We measured fWHR [13] for subjects whose head was turned up to approximately 15° to the side (our analyses are robust to including this amount of turn). Photographs were standardized to a width of 600 pixels and measured using TPSDig v. 2.10 [27]. Additional information on fWHR measurements can be found from the electronic supplementary material, S2.

Individual fitness was calculated as the expected number of children, averaging over whether a father survived the Winter War, i.e. Pr(died in war) × (number of children|died) + Pr(Survived war) × (number of children|survived). Dying in the war was modelled as a logistic regression, with face, regiment, birth place, age and rank as effects (face and age as continuous, others as factors). Similarly, the number of children was modelled as following a Poisson distribution with the same effects, but also with survival as an additional factor, along with an interaction between age and survival. Measurements of face shape were assumed to be normally distributed with the mean set at the true face value, which was used in the analysis. The model was fitted with a Bayesian approach using vague priors (see the electronic supplementary material, S3 for more information).

## 3. Results

**Figure 2.**
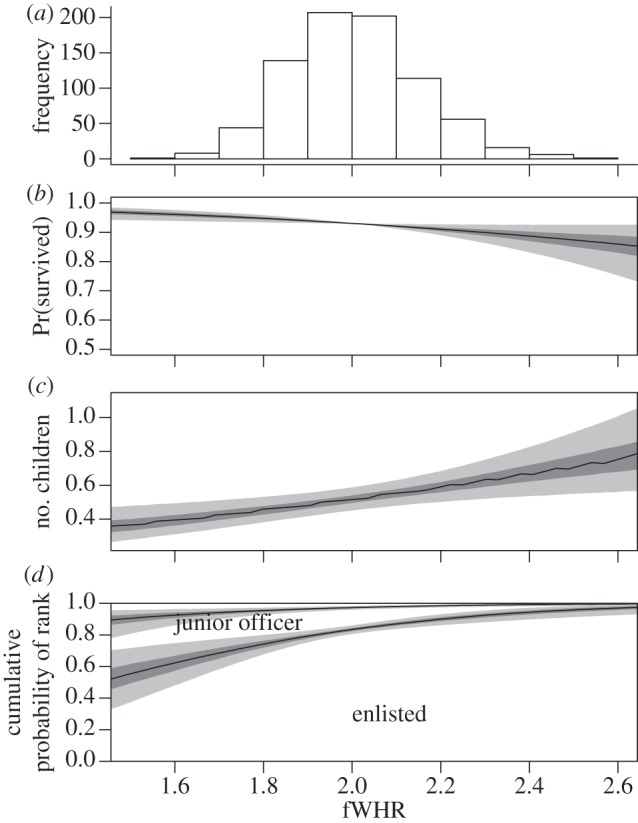
Finnish troops who participated in the Winter War 1939–1940 and the distribution of (*a*) scaled fWHR, (*b*) predicted probability of surviving the Winter War (adjusted so the posterior mode at mean face width is 0.85). Figure contains all 795 individuals, see the electronic supplementary material, S3 for figure with three main regiments only. (*c*) Total number of children for fallen soldiers and (*d*) probability of attaining a rank depending on fWHR. Facial width increases with values (for an enlarged version of figure 2*c* see electronic supplementary material, S4). Dark shaded area represents 50% highest posterior density region and light shaded area represents 95% highest posterior density region.

Initial analysis showed a weak effect of fWHR on survival, (log odds ratio of survival for soldier with face 1 s.d. wider: −0.19, 95% HPDI: −0.37 to −0.04; figure 2*b*; Pr(OR > 0) = 0.007), however, this effect disappeared when only soldiers from the three main regiments were analysed (posterior mode of log odds ratio: −0.014, HPDI: −0.20 to 0.17; Pr(OR > 0) = 0.44). Soldiers with wider faces had more children after controlling for wartime survival, (analysis with full data; a soldier with a face 1 s.d. wider has 1.88 times as many children: 95% HPDI: 1.17–2.88, Pr(Ratio < 1) = 0.0017, figure 2*c*), and males with thinner faces achieved higher rank within the Finnish military prior to the start of hostilities (analysis with full data; log odds ratio of soldier with face 1 s.d. thinner being a higher rank: 0.40, 95% HPDI: 0.19–0.61, figure 2*d*; and see electronic supplementary material, S4).

## 4. Discussion

As fWHR appears to reflect aggressiveness [14–16,20], our finding that LRS increases with facial width is in agreement with evidence that aggression is a sexually selected trait [28]. Human male mating success is correlated with testosterone levels, a trend possibly attributable to increased mating effort [29,30]. It is unlikely that facial width *per se* is under selection: selection operates on a suite of traits, rather than on one single trait [31], and since facial morphology is correlated with several behavioural and physical attributes [6,11,12,15,17,19,20], its evolution is likely to be the effect of pleiotropy. While fWHR did predict LRS, as in previous research the relationship was not a strong one ([24]; figure 2), as might be expected given the amount of other factors that determine LRS. From the perspective of evolutionary biology, fWHR by itself is probably of minor significance; however, it is a useful trait to advance the study of human evolution because of its ease of measurement and its role as an indicator of other behavioural and morphological traits of evolutionary significance.

The result that thinner-faced soldiers achieved higher rank before the start of conflict is somewhat surprising because male social dominance can be predicted by testosterone levels [32], which are presumably higher in wider-faced males. However, dominance in the military may be better predicted by leadership qualities than aggressiveness. The greater trustworthiness [18,19] associated with thinner faces may explain our result that thinner-faced males were able to attain higher rank (and therefore positions of trust) in the military. The military relies on a strict hierarchy, which requires trust and/or fear of punishment to be maintained. In this way, the social structure supports the functioning of the military because individuals who are perceived to be more trustworthy attain higher social dominance, a necessity when leading subordinates into situations of high mortality risk.

In the full data, we found a minor correlation between facial width and survival, however, this effect disappeared when only the three main regiments were analysed, which is a better test of the survival hypothesis. Previous research has found that wider-faced males are less likely to die violent deaths, but only when close physical contact is involved (e.g. death by knife wounds or strangling), and not when technology is used (death by gunshot or poisoning; [16]). Given that technology was involved in wartime mortality, our result is in agreement with this previous work. It is also unclear how alternative tactics (such as avoidance of conflict) might affect overall survival in the entire population. In the case of Finland, mandatory military service for males guaranteed high participation in the Second World War.

An overall picture emerges where positions of trust and power are held by thinner-faced, probably less aggressive, yet possibly more trustworthy individuals. Wider-faced, more aggressive individuals have higher LRS, and the almost equal probability of mortality during the war meant that war did not change this relationship between LRS and fWHR. It is perhaps surprising that aggressive individuals do not achieve any significant fitness or survival benefits from war, however, this may be due to the advent of technology [16]. The fitness outcomes of other conflicts may differ from the case studied here, for example, if an attacking force successfully invades territory and conquering soldiers sire offspring (due to forced copulation or mutual consent) with resident females.
